# Update on non-radiologic ocular and orbital imaging in thyroid eye disease: a review

**DOI:** 10.1117/1.JBO.31.10.100902

**Published:** 2026-10-30

**Authors:** Hannah E. Cho, Christopher D. Yang, Miranda B. An, Gordon T. Kennedy, Bernard Choi, Anthony J. Durkin, Andrew W. Browne, Lilangi S. Ediriwickrema

**Affiliations:** a University of California, Irvine School of Medicine, Irvine, California, United States; b Gavin Herbert Eye Institute, Department of Ophthalmology and Visual Sciences, UC Irvine, Irvine, California, United States; c UC Irvine, Beckman Laser Institute and Medical Clinic, Irvine, California, United States; d UC Irvine, Department of Biomedical Engineering, Irvine, California, United States; e Brunson Center for Translational Vision Research, UC Irvine, Irvine, California, United States

**Keywords:** thyroid eye disease, periorbital imaging, optical coherence tomography, confocal microscopy, spatial frequency domain imaging

## Abstract

**Significance:**

Thyroid eye disease (TED) involves inflammatory, vascular, and fibrotic remodeling of periocular tissues that is traditionally characterized by clinical activity scores and, at times, by conventional neuroimaging. Emerging noninvasive imaging modalities are poised to enhance clinical assessment by objectively quantifying inflammation, vascular alterations, fibrosis, and changes in tissue architecture. These tissue biomarkers have the potential to improve prediction of disease progression and therapeutic response beyond what can be inferred from clinical activity scores alone.

**Aim:**

This review synthesizes established and emerging periocular nonionizing imaging techniques for TED, including ultrasound-based methods, optical coherence tomography (OCT), confocal microscopy (CM), and spatial frequency domain imaging (SFDI).

**Approach:**

Peer-reviewed studies evaluating human subjects with TED were reviewed to understand the technical aspects, advantages, limitations, and clinical relevance of each modality.

**Results:**

Ultrasound elastography and high-frequency ultrasonography enable assessment of tissue stiffness, thickness, and vascularity but are limited by operator dependence and penetration depth. OCT and OCT-angiography provide high-resolution evaluation of retinal, choroidal, and anterior segment structural and vascular changes associated with orbital congestion. Confocal microscopy identifies anatomical alterations that differentiate active from inactive disease. SFDI enables wide-field, noncontact quantification of periocular optical properties, with preliminary findings suggesting sensitivity to inflammatory edema.

**Conclusions:**

This review summarizes current and emerging imaging modalities used to provide quantitative, high-resolution assessment of structural and functional tissue changes in TED.

## 1 Introduction

Thyroid eye disease (TED) is an autoimmune disorder characterized by inflammatory and fibrotic remodeling of orbital tissues, resulting in functional and cosmetic morbidity.^1^ Autoantibody activation of orbital fibroblasts through thyroid-stimulating hormone receptor (TSHR) and insulin-like growth factor-1 receptor (IGF-1R) signaling drives adipogenesis, glycosaminoglycan deposition, and extracellular matrix expansion.^2^ These processes underlie the clinical manifestations of pain, eyelid retraction, proptosis, diplopia, periocular edema, and compressive optic neuropathy (CON).

Although validated clinical scores such as the Clinical Activity Score (CAS), Vision/Inflammation/Strabismus/Appearance (VISA) score and European Group of Graves’ Orbitopathy (EUGOGO) criteria remain the current clinical standard for grading disease activity and severity, they rely on observer-dependent assessments of surface signs and provide limited information about tissue-level changes.^3^ Advances in modalities such as ultrasound elastography, optical coherence tomography (OCT), confocal microscopy, and spatial frequency domain imaging (SFDI) have expanded the research landscape of TED beyond conventional computed tomography (CT) and magnetic resonance imaging (MRI). These newer modalities are noninvasive, radiation-free, and capable of resolving functional and biomechanical tissue properties—such as stiffness, microstructure, and optical/hemodynamic characteristics—at a level of detail relevant to active tissue remodeling. As such, they offer promising tools for capturing the tissue-level disease activity that clinical activity scores and conventional imaging may fail to detect. This review integrates findings across established and emerging imaging platforms to highlight technological advances, methodological challenges, and translation opportunities in TED research.

## 2 Methods

### 2.1 Literature Search

A targeted literature search was conducted on PubMed and Google Scholar for studies published between 2000 and 2026 using keywords “thyroid eye disease” combined with modality-specific terms (ultrasound, confocal, OCT, and SFDI). Studies were included if they 1. evaluated human subjects with TED and reported outcomes relevant to diagnosis, disease staging, or management, or 2. described technical validation or feasibility of imaging modalities with application in TED. These articles were reviewed by three independent study members. Articles evaluating nonhuman subjects or not written in English were excluded. This process yielded 26 articles that met criteria for further review and analysis.

## 3 Discussion

### 3.1 Ultrasound

Conventional computed tomography (CT) and magnetic resonance imaging (MRI) remain the primary imaging modalities for evaluating extraocular muscle (EOM) and orbital fat expansion in TED. However, CT is limited by ionizing radiation, while MRI is costly and may not be readily available for serial monitoring.^4^ Ultrasound-based techniques may complement conventional imaging by providing quantitative measures of tissue stiffness that could help distinguish inflammatory changes from chronic fibrosis, assess the risk of disease progression, and predict response to immunomodulatory therapy. Ultrasound has also been used to characterize EOM in pediatric patients with thyroid dysfunction.^5^

High-frequency ultrasonography (HFUS), which applies ultrasound frequencies >10  MHz, provides high resolution for superficial tissues such as skin, subcutaneous fat, and blood vessels (Table 1).^6^ HFUS can precisely determine tissue thickness when paired with color Doppler, which enables real-time evaluation of vascular changes, which may be useful in TED management.

**Table 1:** Description of current clinical applications of high-frequency ultrasound (HFUS) in the periocular region.

Clinical application	Frequency	Depth	Frame rate/resolution
Image-guided Facial Rejuvenation Procedures^7^	15 MHz	2 cm	50 frames/s
Monitoring of TED-related Lid Retraction and Proptosis^7^	15 MHz	2 cm	50 frames/s
Visualization of Subcutaneous Lesions^8^	20 to 100 MHz	1.5 mm	16 μm

Ultrasound elastography (USE) is an extension of conventional ultrasonography that assesses tissue elasticity and stiffness by measuring the degree of tissue displacement or speed of sound wave propagation through tissue and is useful in detecting pathological structural changes in tissue.^9^ Quantitative assessment of tissue stiffness has been proposed as a means of distinguishing active inflammatory changes from chronic fibrosis in TED.^4^ Elastography has shown particular promise in quantitatively distinguishing inflammation from fibrosis, which may help differentiate the active and chronic phases of TED.^10^

Two main types of USE are commonly used: strain elastography (SE), which evaluates tissue deformation in response to manual compression and provides a semi-quantitative readout, and shear wave elastography (SWE), which evaluates absolute tissue stiffness by generating automated acoustic pulses, yielding a more objective assessment (Fig. 1).^11^

**Fig. 1:**
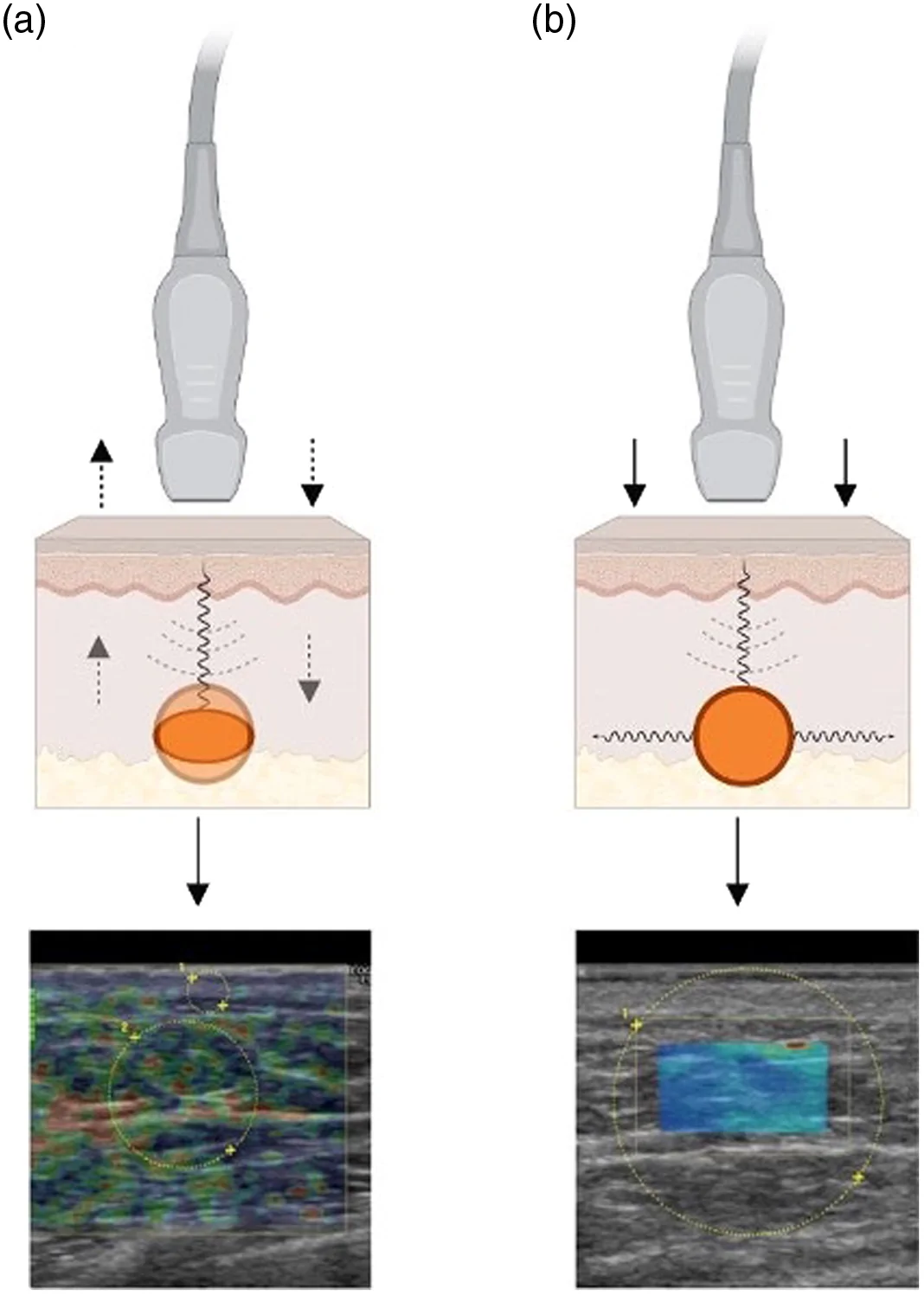
Schematic representation of (a) strain elastography—the tissue of interest is manually compressed, and the amount of deformation relative to the surrounding tissue is measured; (b) shear-wave elastography—shear waves generated through the transducer propagate laterally, distal to the region of interest, allowing the user to visualize tissue stiffness as a color-coded map in two dimensions.

SE provides semi-quantitative information by generating stiffness maps that compare tissue deformation during and after manual compression. In contrast, SWE directly quantifies tissue stiffness by measuring shear wave propagation through tissue using high-frequency linear ultrasound transducers at a frequency of 10 to 20 MHz, with stiffness typically reported as Young’s modulus in kilopascals (kPa).^12^ SWE offers sub-millimeter spatial resolution with a penetration depth of several centimeters.^13^ In TED, inflammatory cell infiltration and edema during the active phase, as well as progressive fibrosis of the EOM and retrobulbar adipose tissue during the inactive phase, contribute to increased tissue stiffness, although fibrosis is associated with the highest stiffness values.^10^ Guazzaroni and colleagues demonstrated the ability of SWE to assess stiffness of the optic nerve, chorioretinal complex, and periorbital region in patients with glaucoma, making it a candidate tool to detect pathological tissue alterations in TED.^14^ Beyond diagnosis, quantitative SWE measurements may also provide prognostic value, with one study showing that elevated stiffness despite low CAS may reflect underlying fibrosis rather than active inflammation and may predict lower likelihood of response to certain immunomodulatory therapy.^10^ Furthermore, SWE operation is automated, which reduces the risk of operator dependency and challenges associated with manual scanning.

SE has likewise been used to characterize orbital tissue biomechanics in TED.^15^^,^^16^ Using both SE and SWE, Ozdemir et al. identified significant alterations in the stiffness of the medial rectus muscle and optic nerve in patients with Graves’ disease without clinically apparent ophthalmopathy. SE demonstrated a significant increase in medial rectus elasticity, while SWE detected lower mean elastic modulus values in both the medial rectus muscle and optic nerve compared with healthy controls, suggesting that ultrasound elastography may be capable of detecting subclinical edema in early TED before clinically evident disease develops and therefore may be useful as a prognostic tool. However, these findings should be interpreted in the context of the study population, which consisted of patients with Graves’ disease without clinically apparent ophthalmopathy. Direct comparison of studies evaluating the use of ultrasound elastography in distinguishing established active and inactive TED is limited.

Furthermore, implementation of SE as a routine imaging modality for TED is still precluded by an absence of dedicated ocular transducers and challenges in obtaining adequate mechanical compression of deeper orbital and intraocular tissues. The quality of the current body of evidence remains restricted by small sample sizes, cross-sectional study designs, and a lack of standardized acquisition protocols and validated diagnostic thresholds across studies.

Overall, the noninvasive, cost-effective, and portable nature of US makes it readily accessible in the clinical setting. Ultrasonography does have notable limitations, nonetheless, such as operator dependence and lack of specificity and sensitivity in providing high-resolution detail regarding TED-related tissue edema, disease activity, and fibrosis.^17^

### 3.2 Optical Coherence Tomography

Optical coherence tomography (OCT) is a noninvasive imaging technique that provides high-resolution *in vivo* cross-sectional images of the retina, choroid, and optic nerve.^18^ By utilizing infrared light, OCT achieves significant resolution 3 to 100 times greater than ultrasound, making it ideal for detailed microstructural assessment of TED (Table 2). In TED, this resolution advantage is particularly useful for detecting consequences of CON: OCT can quantify peripapillary retinal nerve fiber layer (RNFL) and macular ganglion cell layer (GCL) thickening, reflecting early axoplasmic stasis from optic nerve compression, followed by thinning that can precede perimetric visual field changes.^19^ OCT also reveals subfoveal choroidal thickening due to venous outflow compression from orbital inflammation and EOM enlargement.^20^ Because these structural changes can occur before clinically apparent visual field loss, spectral-domain OCT (SD-OCT) measurements may serve as early, quantitative markers of disease activity and treatment response. Its speed and wide availability at point-of-care in ophthalmology clinics further make it well suited for serial monitoring of at-risk patients.

**Table 2:** Description of OCT types and clinical application in TED. SFCT: subfoveal choroidal thickness; CVI: choroidal vascularity index.

OCT	Description	Applications
Spectral-domain OCT (SD-OCT)^24^	High axial resolution for detailed structural assessment of intraocular and periocular tissues; most used OCT modality	Enhanced depth imaging of choroidal vasculature indices (e.g. SFCT and CVI), tracks TED progression and activity
Swept-source OCT (SS-OCT)^25^	Greater penetration depth than SD-OCT, greater sensitivity and lower signal-to-noise ratios, faster scanning speeds, more expensive than SD-OCT	Reliably measures CVI; imaging deeper layers enhances accuracy
High-definition OCT^24^	Used with SS-OCT and SD-OCT to image choroidal structures	Evaluates CVI
OCT angiography^26^	Noninvasive microvascular imaging of the retinal vasculature and optic nerve	Detects active and inactive TED, but analyzes perfusion density in various retinal layers; correlates with TED severity

Spectral-domain OCT (SD-OCT) is particularly useful for visualizing the three-dimensional microstructure of the retinal cellular layers and choroidal vascular beds that may reflect changes in active TED (Fig. 2).^21^ Characteristic OCT findings seen in active TED include increased RNFL or GCL thickness and subfoveal choroidal thickness (SFCT).^22^^,^^23^

**Fig. 2:**
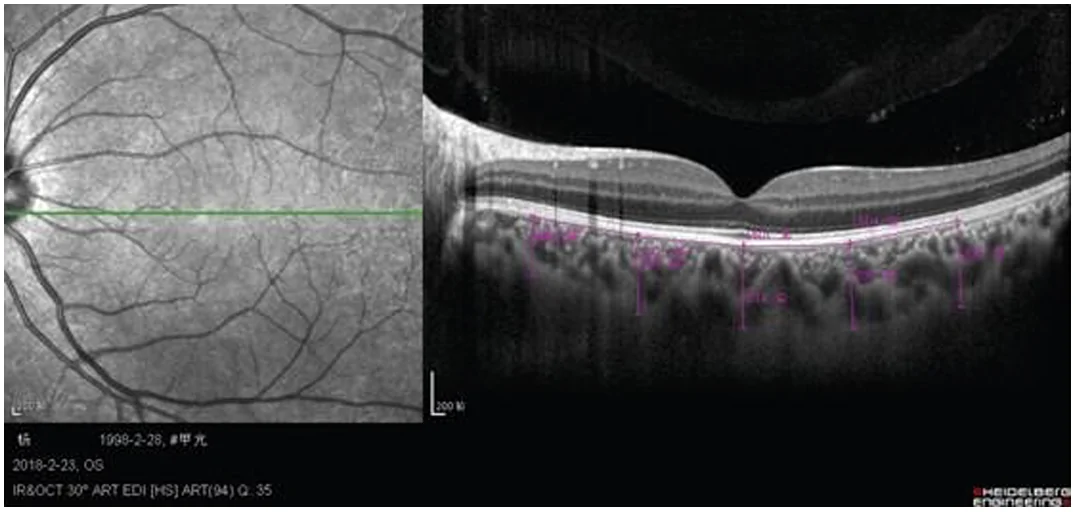
Representative spectral-domain OCT image demonstrating pachychoroid (purple) in an eye with active TED. Adapted from Yu et al.,^20^ under the Creative Commons Attribution (CC BY) license.

A recently published observational study found SFCT was lower in patients with higher CAS,^26^ but these findings contrasted with those from another study of swept-source OCT (SS-OCT) that found a significant positive correlation between choroidal thickness and CAS.^27^ Gurkan et al. evaluated changes in the retinal nerve fiber layer (RNFL) and optic nerve sheath diameter (ONSD) in patients with TED, finding that RNFL thickening positively correlated with ONSD, even for patients with inactive disease.^28^ This finding suggests that RNFL thickening may indirectly reflect the degree of orbital congestion found in chronic TED, and increased intraorbital pressure driven by active TED may compress the optic nerve, resulting in hypoperfusion or axoplasmic stasis.

SS-OCT, with greater resolution for visualizing subretinal tissues, more clearly highlights and recapitulates the choroidal changes seen in SD-OCT (Fig. 3) and further expands the role of OCT in TED with high-resolution imaging of microstructural changes in the anterior segment. Gün et al. demonstrate that both the length and area of Schlemm’s canal (SC) are significantly reduced in eyes with TED compared with control eyes, suggesting that TED-related orbital congestion may impair aqueous humor outflow and contribute to elevated intraocular pressure.^29^

**Fig. 3:**
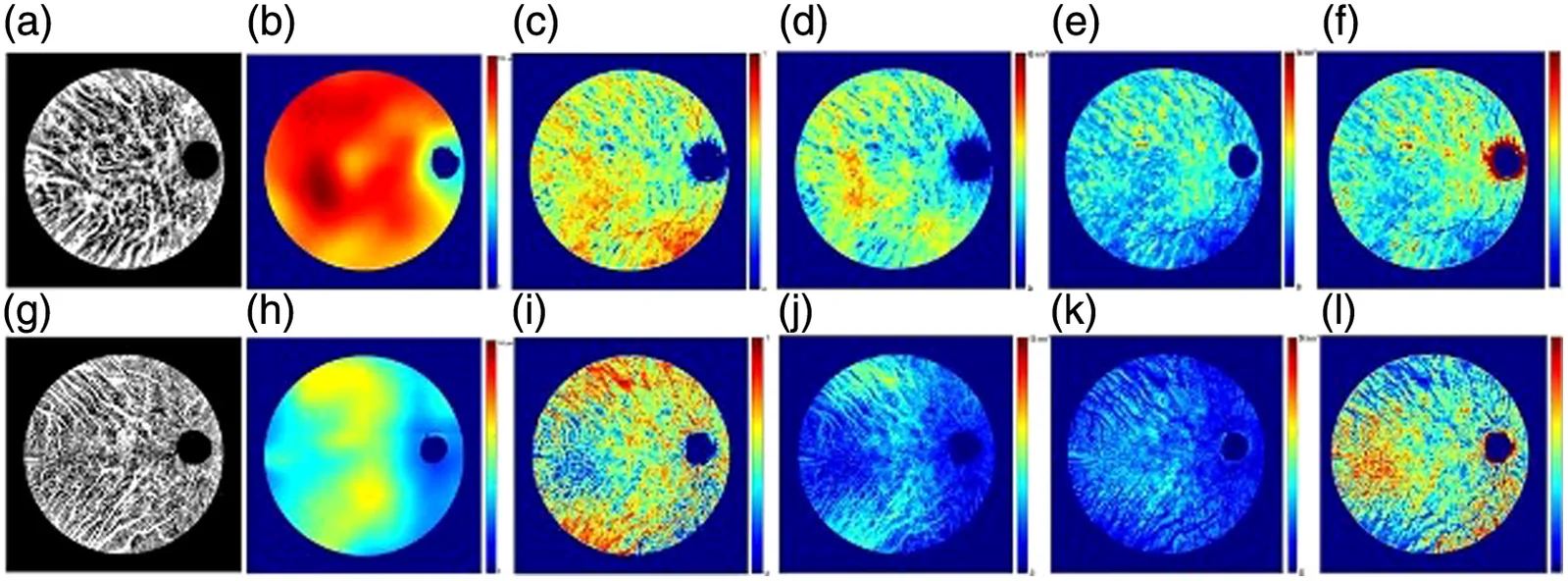
Representative *en face* swept-source OCT images demonstrating increased (red) choroidal thickness (b, h), vascularity (c, i), vascular volume (d, j), mesenchymal thickness (e, k), and mesenchyme-vessel ratio (f, l) in an eye with active TED (a–f) compared with a control eye (g–l). Adapted from Dai et al.,^30^ under CC BY license.

Of note, Gün et al. did not find a significant correlation between Schlemm’s canal dimensions and CAS, disease duration, or RNFL thickness, suggesting these structural changes may occur independently of TED disease activity. Nevertheless, SS-OCT offers a non-invasive and high-resolution modality to assess anterior segment structures that may complement posterior segment and orbital interrogation in TED. The lack of longitudinal data and small sample sizes underscore the need for further research to elucidate the temporal relationship between SC measurements and TED progression.

OCT is limited to imaging optically accessible tissues with a penetration depth of two millimeters.^18^^,^^31^ These features restrict its ability to evaluate deeper orbital structures relevant to TED, such as EOMs and orbital fat.^32^ Because of this, details observed with OCT are likely best a complementary resource to mainstay imaging modalities used in comprehensive TED management.

Visualizing changes in retinal blood flow caused by orbital compression in TED offers another metric for detecting changes in disease activity. OCT angiography (OCTA) is a non-invasive imaging modality that allows *in vivo* visualization of three-dimensional retinal vascular anatomy. TED has been linked to reduced peripapillary retinal perfusion on OCTA (Fig. 4), which is thought to reflect orbital congestion and impaired optic nerve perfusion.^33^ Although these vascular changes have been associated with CON, current evidence does not establish a causal relationship, and reported OCTA biomarkers remain heterogeneous across disease stages.

**Fig. 4:**
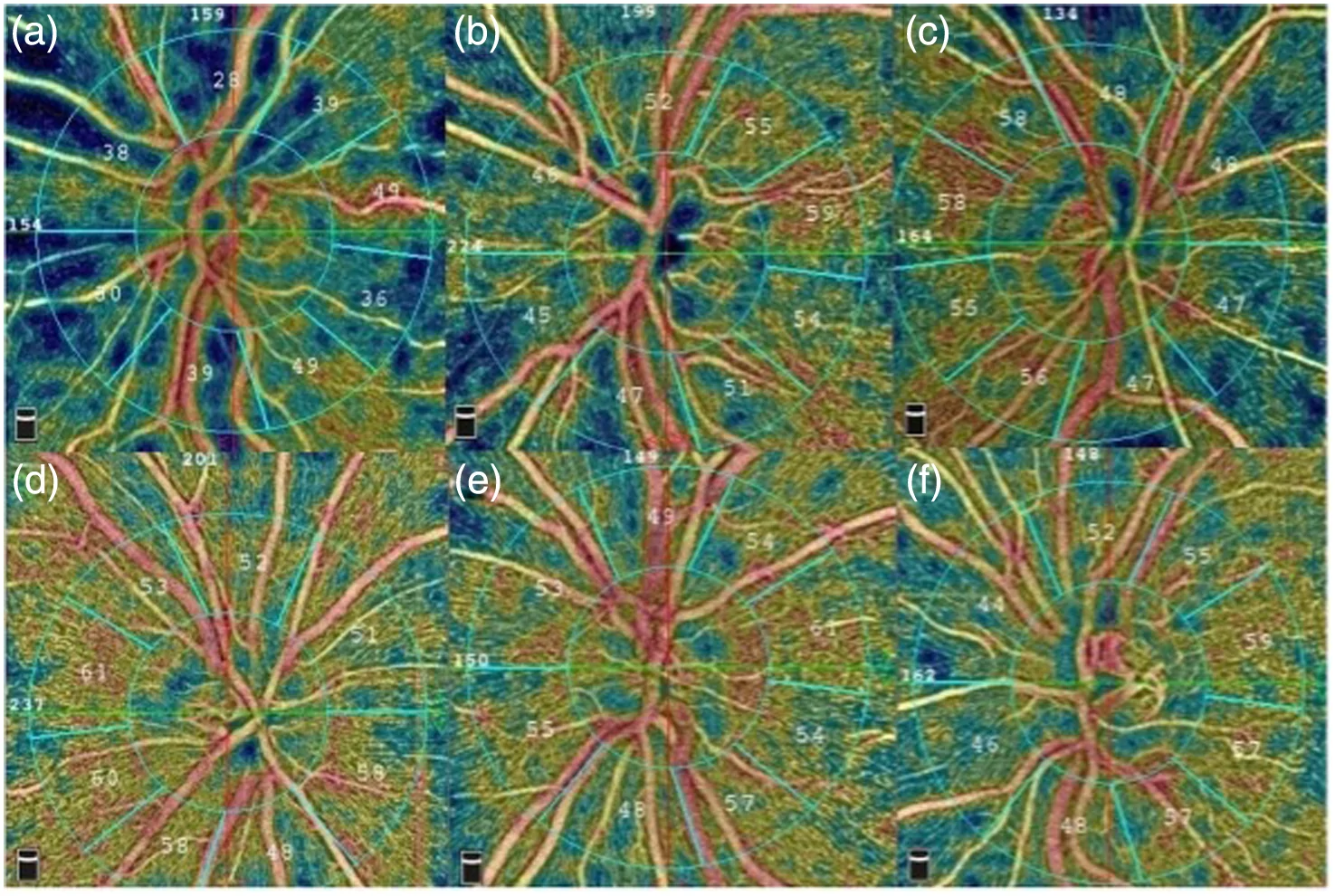
Representative *en face* OCTA images at the level of the peripapillary capillary network demonstrating flow deficits (blue) in eyes with active TED without compressive optic neuropathy or vision loss (a–c) compared with age-matched controls (d–f). Each image captures a 4.5×4.5  mm area of the retinal vascular plexus. Adapted from Sabermoghaddam et al.,^34 ^under CC BY license.

Sun and colleagues used OCTA to visualize retinal vascular changes and found that patients with active TED have a significantly thicker choroid and higher choroidal vascularity index (CVI) compared with those with inactive TED.^35^ Interestingly, these observed choroidal changes correlated more strongly with the degree of proptosis than CAS. Moreover, color doppler imaging revealed elevated retrobulbar blood flow in the posterior ciliary artery (PCA), with increased peak systolic velocity (PSV) and end diastolic velocity, as well as lower resistance index and pulsatile index. Among these parameters, the PSV of the PCA shows an independent association with CAS, suggesting its potential as a marker for TED activity. A separate study focusing on microvascular changes in the peripapillary region found that active TED was associated with higher choroidal vascular indices than inactive disease, suggesting again that TED-mediated inflammation triggers a hypervascular process along the posterior segment.^33^ These findings emphasize the effects of orbital congestion on posterior segment perfusion in TED and underscore the diagnostic utility of vascular indices in monitoring disease activity.

In summary, there are discrepancies in the anatomical and vascular findings identified by OCT and OCTA in TED, particularly regarding choroidal thickness and correlation of this metric with disease activity. Some studies have reported a positive correlation between subfoveal choroidal thickness and active TED, suggesting that choroidal thickening reflects orbital congestion and disease activity. However, other studies have found the opposite, challenging the reliability of choroidal thickness as a marker of active TED. Studies of OCTA have revealed that choroidal thickness and stromal volume negatively correlate with CON, while choroidal vascular indices positively correlate, further complicating the interpretation of choroidal metrics across disease stages. These discrepancies underscore the need for standardized imaging protocols and larger, longitudinal studies to clarify the utility of OCT and OCTA biomarkers in TED.

### 3.3 Confocal Microscopy

Confocal microscopy is an imaging modality that utilizes focused light to produce high-resolution cross-sectional images of living tissue.^36^ The most commonly reported use of confocal imaging in TED is the assessment of meibomian gland disease as a proxy for TED exacerbation.^37^ Although confocal findings in TED overlap with those observed in meibomian gland dysfunction (MGD), active TED demonstrates more prominent inflammatory features, including acinar wall irregularity, periglandular interstitial heterogeneity, and inflammatory cell infiltration,^38^ whereas primary MGD is more typically characterized by ductal obstruction, gland dropout, and increased meibum reflectivity. These distinctions suggest that confocal microscopy may help differentiate inflammatory ocular surface disease secondary to TED from age-related or primary MGD, offering a potential noninvasive biomarker for detecting and monitoring ocular surface inflammatory activity in TED patients. Cheng and colleagues report that active TED is associated with a greater degree of acinar irregularity, acinar wall inhomogeneity, and peri-glandular interstitial inhomogeneity, all of which reflect active inflammation.^39^ Conversely, inactive TED is associated with increased meibum secretion reflectivity and severe MG fibrosis, both of which are indicative of chronic obstruction and fibrosis.^39^ Both active and inactive TED groups display lower MG acinar density and orifice area compared with controls; metrics like these may prove useful as monitoring tools that differentiate TED disease stages and guide management. Yet, despite its high spatial resolution, *in vivo* confocal microscopy remains primarily a research tool. Examination typically requires approximately 10 to 20 min per eye, visualizes only a limited number of meibomian glands at one time, and requires experience for image acquisition and interpretation. Furthermore, diagnostic thresholds and standardized acquisition protocols have not been established, limiting widespread clinical adoption. Future studies are needed to determine the sensitivity and reproducibility of confocal biomarkers for monitoring disease progression and treatment response in TED.

### 3.4 Spatial Frequency Domain Imaging

Spatial frequency domain imaging (SFDI) is a wide-field imaging method that uses spatially modulated visible and near-infrared light to interrogate subsurface tissue to separate optical absorption ($\mu_{a}$) from optical scattering ($\mu_{s}^{\prime }$) effects and shows promise as a noninvasive tool for managing TED. Scattering-derived measures, specifically, may reflect edema, cellular infiltration, or early fibrotic change. Our group has published a facial phantom study^40^ validating SFDI measurement accuracy along the curved surfaces of the periocular region and has published a manuscript^41^ detailing normative human subject periocular SFDI values, establishing a baseline against which pathological changes in TED can be compared. All subjects gave their informed consent to take part, and the study followed a protocol cleared by UC Irvine’s Institutional Review Board for research involving human participants (protocol HS#2019-2954). Given its rapid, noncontact, radiation-free acquisition, SFDI holds potential for longitudinal monitoring of inflammatory activity and therapeutic response in TED, though clinical validation remains early-stage.

The Reflect RS™ (Modulim, Inc., Irvine, California, United States) specifically performs SFDI by measuring calibrated diffuse reflectance images over a $15\times 20\;{\mathrm{cm}}^{2}$ field of view, with a pixel resolution of ∼0.5  mm. Spatially modulated light from eight light-emitting diodes (LEDs), with center wavelengths from 470 to 851 nm, is projected onto the tissue at five to seven evenly spaced spatial frequencies from 0 to 0.2/mm. Subjects are stabilized using a chin rest with dual forehead apposition to minimize motion artifacts, and periocular regions of interest are imaged (inferior temporal quadrant (ITQ), inferior nasal quadrant (INQ), and control-forehead (FH)).

Although SFDI has been applied in a range of clinical and preclinical settings, including the assessment of laser treatment efficacy for port wine birthmarks,^42^ wide-field cerebral hemodynamic imaging in rodents, and skin involvement in systemic sclerosis (scleroderma), our group focuses on its potential in TED management. We evaluated SFDI’s accuracy in measuring periocular optical properties using a solid tissue-simulating facial phantom and observed minimal variability with repeated measurements along the periocular region [Figs. 5(a)–5(c)].^40^

**Fig. 5:**
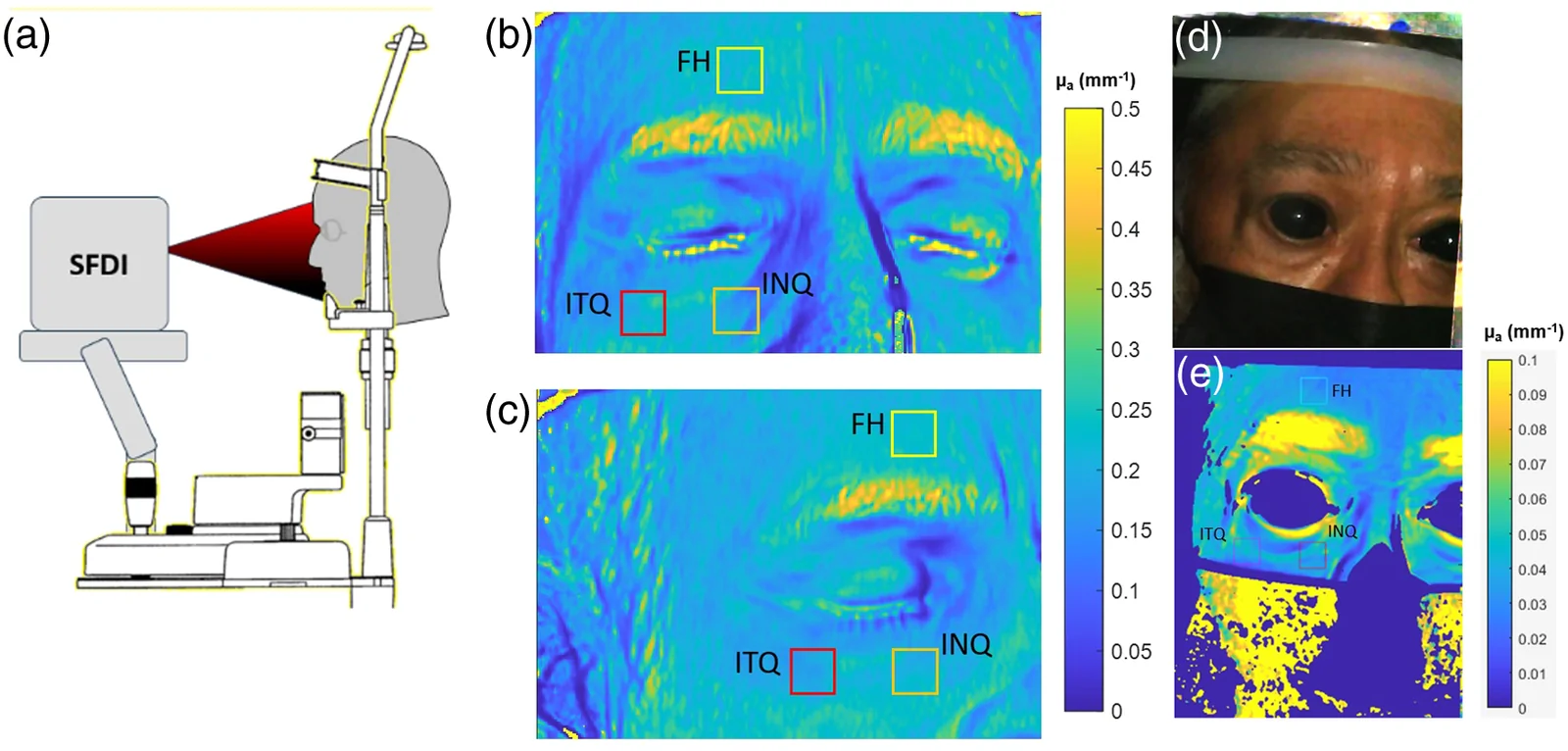
(a) SFDI system consisting of a light source projecting spatially modulated light onto a facial phantom positioned in a chin rest. Representative human subject was imaged with a resultant (b) “en face” absorption coefficient map and (c) “side profile” absorption coefficient map, with identified regions of interest (ROI): inferior temporal quadrant (ITQ), inferior nasal quadrant (INQ), forehead (FH), used to measure reduced scattering ($\mu_{s}^{\prime }$) and absorption ($\mu_{a}$) coefficients. Absorption coefficient map of (d) a patient with active thyroid eye disease wearing a face-mask (e) with identified ROI: ITQ, INQ, and FH. Adapted in part from Ediriwickrema et al.^41^

Our ongoing studies aim to identify cutaneous biomarkers that reflect periocular congestion and erythema, which are indicative of active TED. Preliminary unpublished data from three active type II TED patients reveal decreased periocular reduced scattering coefficient values (mean $\mu_{s}^{\prime }$ 0.98, 1.16, and $1.26\;{\mathrm{mm}}^{-1}$ for ITQ, INQ, and FH, respectively), as compared with 25 healthy normal subjects (mean $\mu_{s}^{\prime }$ 1.21, 1.36, and $1.44\;{\mathrm{mm}}^{-1}$ for ITQ, INQ, and FH, respectively), potentially due to soft tissue edema and active inflammation [Figs. 5(d)–5(e)]. Prior work by our group has demonstrated that SFDI-derived optical property metrics, including reduced scattering coefficient and tissue oxygenation, can capture microstructural remodeling and inflammatory changes in burn models.^43^^–^^45^ These preliminary findings motivate larger, adequately powered studies to determine whether SFDI-derived optical properties track TED activity. As a portable, noncontact device, SFDI is well-suited for clinical application, offering a promising new approach to monitoring and managing TED progression.^46^

## 4 Conclusion

Periocular imaging in TED is rapidly moving beyond clinical exam scores toward multidimensional, quantitative characterization of tissue architecture and function, leveraging the complementary strengths of various modalities. Rather than replacing clinical activity scores, emerging imaging biomarkers may provide orthogonal information regarding the biological state of orbital tissues, enabling more precise phenotyping of TED, improving prognostication, and guiding personalized treatment programs. Although conventional imaging techniques such as CT and MRI remain informative, optical and acoustic modalities offer objective high-resolution quantification of biometric changes related to inflammation and tissue remodeling. High-resolution systems such as OCT and CM provide visualization of microstructural detail within limited fields of view, such as retinal/choroidal changes or meibomian gland abnormalities. Conversely, SFDI has the potential to stand out by enabling wide-field, noncontact, quantitative mapping of periocular tissue optical properties, allowing for interrogation of subsurface tissue volumes several millimeters in depth. Preliminary findings suggest that SFDI-derived metrics, including the reduced scattering coefficient ($\mu_{s}^{\prime }$), may be sensitive to periocular soft tissue edema in active TED, although this observation rests on a small number of patients and requires confirmation in a larger cohort. Although advancements introduce new quantitative biomarkers, such as shear-wave velocity and choroidal vascularity index, variability in acquisition protocols and outcome measures across these platforms currently limits widespread clinical adoption. Ultimately, integrating structural imaging with quantitative optical and photonic tools may bridge the gap between anatomical visualization and functional tissue characterization, advancing both the mechanistic understanding of TED pathophysiology and its clinical management.

## Data Availability

Data sharing is not applicable to this article, as no new data were created or analyzed in this review.
